# The Multi-Kingdom Microbiome of Wintering Migratory Birds in Poyang Lake, China

**DOI:** 10.3390/v16030396

**Published:** 2024-03-03

**Authors:** Jia Liu, Xiyan Li, Wentao Song, Xiaoxu Zeng, Hui Li, Lei Yang, Dayan Wang

**Affiliations:** 1 National Institute for Viral Disease Control and Prevention, Chinese Center for Disease Control and Prevention, Beijing 102206, China; liujia@cnic.org.cn (J.L.); lixiyan@cnic.org.cn (X.L.); zengxiaoxu@cnic.org.cn (X.Z.); yanglei@cnic.org.cn (L.Y.); 2 School of Public Health, Xiamen University, Xiamen 361005, China; songwentao99@126.com; 3 Nanchang Center for Disease Prevention and Control, Nanchang 330038, China; nccdclih@163.com

**Keywords:** Poyang Lake, migratory birds, metagenomic, virome, public health, taxonomic biomarker

## Abstract

Wild birds are a natural reservoir for zoonotic viruses. To clarify the role of migratory birds in viruses spread in Poyang Lake, we investigated the microbiome of 250 wild bird samples from 19 species in seven orders. The bacterial and viral content abundance and diversity were preliminarily evaluated by Kraken2 and Bracken. After de novo assembly by Megahit and Vamb, viral contigs were identified by CheckV. The reads remapped to viral contigs were quantified using Bowtie2. The bacterial microbiome composition of the samples covers 1526 genera belonging to 175 bacterial orders, while the composition of viruses covers 214 species belonging to 22 viral families. Several taxonomic biomarkers associated with avian carnivory, oral sampling, and raptor migration were identified. Additionally, 17 complete viral genomes belonging to *Astroviridae*, *Caliciviridae*, *Dicistroviridae*, *Picornaviridae*, and *Tombusviridae* were characterized, and their phylogenetic relationships were analyzed. This pioneering metagenomic study of migratory birds in Poyang Lake, China illuminates the diverse microbial landscape within these birds. It identifies potential pathogens, and uncovers taxonomic biomarkers relevant to varied bird habitats, feeding habits, ecological classifications, and sample types, underscoring the public health risks associated with wintering migratory birds.

## 1. Introduction

Birds are important hosts for a wide range of pathogens and sources of emerging infectious diseases in humans. Wild birds carry zoonotic viruses such as avian flu, avian paramyxovirus serotype 4, and other avian viruses that not only infect poultry but also pose substantial threats to human health [1,2]. During migration, birds traverse diverse regions, potentially introducing various pathogens, particularly viruses, into new areas. Interactions between migratory birds and backyard poultry—sharing food, water sources, and habitats—significantly elevate the risk of human exposure to avian viruses.

Poyang Lake, located in China, is the largest freshwater lake in the country and serves as a crucial stopover for migratory birds during their journey across the East Asian-Australasian Flyway. Generally, from October to March of the following year, migratory waterbirds from Siberia and Northeast Asia winter at Poyang Lake [3]. During this period, the lake experiences its dry season, resulting in a reduced water area predominantly composed of herbaceous meadows, mudflats, and isolated water bodies, facilitating pathogen spread [4,5].

Metagenomic analysis has emerged as a powerful tool for identifying and characterizing viral communities in various samples [6,7]. This approach enables the detection of a wide range of viruses, including those challenging to culture using traditional methods, by sequencing genetic material from complex microbial communities. Studies employing metagenomic sequencing have extensively explored viromes in human and animal fecal samples, soil, water, and even air samples, revealing the intricate diversity of viral communities in different ecosystems [8,9,10,11]. However, there is a significant gap in the detection and exploration of viromes in wild bird populations, as the current focus remains primarily on human and domesticated animal samples, leaving wild avian populations underrepresented.

Our study aimed to assess the microbiome diversity in various migratory bird species at Poyang Lake by analyzing their bacterial and viral communities. Additionally, we investigated the taxonomic and ecological associations between avian hosts and their respective microbiomes. The primary focus was on using metagenomic analysis to explore the potential risks associated with viromes carried by migratory birds and their potential transmission pathways to wildlife and humans. Furthermore, we underscored the importance of the correlation between bacterial and viral communities, and we identified several complete viral genomes. This highlights the critical significance of adopting the One Health approach to comprehensively address the complex interconnections among human, animal, and environmental health.

## 2. Materials and Methods

### 2.1. Wetland Location and Sample Collection

Poyang Lake (28°11′–29°51′ N, 115°49′–116°46′ E) is the largest freshwater lake in China at the junction of the middle and lower reaches of the Yangtze River, and it is an important stopover site for migratory wild birds of the East Asia–Australia bird migration route during the autumn and winter seasons [12,13].

Fecal, pharyngeal swab, and cloacal swab samples were gathered from Duchang Tangkou Wildlife Rescue Station (DC, 29.209982 N, 116.463861 E) and Jiangxi State-owned Henghu Comprehensive Reclamation Farm (HH, 28°50′–29°03′ N, 116°04′–116°10′ E) between 27 February 2019, and 10 March 2019. In total, 253 samples were collected, with successful sequencing achieved for 250 of them. These sequenced samples encompass 19 species from 7 orders: *Ciconiiformes* (14), *Gruiformes* (3), *Charadriiformes* (17), *Podicipediformes* (18), *Falconiformes* (2), *Strigiformes* (8), *Anseriformes* (188) (Table S1). All samples were immediately placed in individual sterile tubes at 4 °C and transported directly to the laboratory for storage at −75 °C.

### 2.2. Sample Grouping Strategy

Based on avian ecological factors, feeding habits, sample types, and taxonomic orders, we conducted an analysis of the bacterial microbiome and virome composition and diversity at various taxonomic levels (Figures S1 and S2). We have implemented two grouping strategies, where we categorize 250 samples based on host characteristics or sample type, respectively. Our classification included five subgroups based on the hosts’ feeding habits and ecological classifications: Natatores-Carnivorous, Natatores-Herbivorous, Natatores-Omnivorous, Raptor-Carnivorous, and Wader-Carnivorous, which we named Grouping type 1. Another classification, named Grouping type 2, included two subgroups based on the sample types: feces-like sample and pharyngeal swab sample. The feces-like sample subgroup consisted of cloacal swab samples and fecal samples.

### 2.3. Nucleic Acid Extraction and cDNA Preparation

The total RNA of each sample was extracted using an RNA extraction kit (magnetic bead method) (MatriDx Biotech Corp., Hangzhou, China, Cat# MD005T) according to the manufacturer’s instructions. The RNA was eluted in a final volume of 70 µL of RNase-free water.

An RNA reverse transcription kit (Matridx Biotech Corp., Hangzhou, China, Cat# MD017) was used for cDNA preparation. For each sample, 14 µL of RNA was mixed with 2 µL of the enzyme mixture in buffer in a 0.2 mL tube of as the first step. The total reaction volume was 20 µL. Reverse transcription was performed using the Bio-Rad T-100 cycler (Hercules, CA, USA). The reaction conditions were as follows: 25 °C for 10 min, 50 °C for 30 min, 75 °C for 10 min, and held at 4 °C. The chain synthesis products (20 µL) were added to 2 µL of the enzyme mixture in the buffer and 18 µL nuclease-free ddH2O in the second step. A total volume of 40 µL was used in the second-strand cDNA synthesis. The reaction conditions were as follows: 16 °C for 15 min and stored at 4 °C. DNA purification was performed using a DNA purification kit (magnetic bead method) (Matridx Biotech Corp., Hangzhou, China, Cat# MD012T). The 35 µL purification solution was prepared for the follow-up experiments. The cDNA concentrations were determined using a Qubit X-Green II dsDNA Quantitation Kit (Yuheng Biotech Corp., Suzhou, China, Cat# Q2038). All cDNA was diluted 1:200 with a dsDNA HS buffer.

### 2.4. Metagenome Sequencing

Libraries were constructed according to the manufacturer’s protocol using the Metagenomic DNA Library Preparation Kit (MatriDx Biotech Corp., Hangzhou, China, Cat# MD001T). DNA fragmentation, adaptor addition, purification, quantification, and pooling were conducted as per the manufacturer’s instructions. Sequencing was carried out using a NextSeq500 sequencer (75 bp single end). The obtained raw reads were filtered for quality and complexity to obtain clean raw reads. Sequencing procedures were performed by MatriDx Biotech (Hangzhou, China).

### 2.5. Bioinformatic Analysis and Phylogenetic Analysis

For each library, sequencing reads were trimmed using the Trimomatic v0.33 [14]. The obtained reads were used to perform taxonomic analysis through the kraken2 program with default parameter settings using the Standard PlusPF database (https://benlangmead.github.io/aws-indexes/k2, accessed on 17 May 2021) [15]. The species abundance after classification with kraken2 was re-estimated using Bracken (Bayesian Reestimation of Abundance after Classification with Kraken2) [16]. No filtering of host/bacterial reads was performed before taxonomic analysis.

The downstream data analysis of the kraken2 and bracken results was conducted using the R package “microeco” [17,18]. To account for potential influences of sequencing depth on community diversity, the OTU table was rarified to standardize all samples to 10,000 reads. Species richness and alpha diversity were assessed using the Chao1 index. To evaluate the significance of differences in alpha diversity and taxon abundance among different groups, ANOVA tests were performed. Random Forest analysis was employed to identify important indicator taxa using the class trans_diff method, utilizing 1000 trees, a significance threshold value of 0.01, and default settings. To mitigate the influence of rare taxa, less abundant taxa (average abundance < 0.01% in all samples) were excluded from the Random Forest analysis. The abundance of biomarkers was assessed using LEfSe in the class tran_diff with default parameters. Bray–Curtis dissimilarity was calculated to assess the distances between each pair of groups. Principal Coordinate Analysis (PCoA) was then conducted to visualize the differences among groups using the class trans_beta method.

Host and rRNA reads were removed by bowtie2 [19]. Each sample was individually assembled using MEGAHIT v1.2.9 with default parameters [20]. Genes were predicted by Prodigal V2.6.3 [21]. The complete genes were searched against the Nr database with the e-values 1e-5 by BLASTp (https://blast.ncbi.nlm.nih.gov/Blast.cgi, accessed on 18 January 2024). We combined the viral sequence with the results identified by Vamb [22]. CheckV was used to identify the completeness of viral genomes [23]. RNA-dependent RNA polymerase (RdRp) regions of viral genes and the closely related amino acid sequences were aligned by MAFFT v7.222 [24]. A phylogenetic tree was constructed using IQ-TREE 2 based on the maximum-likelihood method and the ultrafast bootstrap value was tested with 1000 replications [25,26]. The tree was finally edited and visualized in the interactive Tree of Life (iToL) [27].

### 2.6. Nucleotide Sequence Accession Numbers

The clean data and bio-samples can be found in BioProject PRJCA022325 of the National Genomics Data Center (https://ngdc.cncb.ac.cn/, accessed on 18 January 2024). The complete genome sequences of viruses identified in our study were submitted to GenBase and assigned the accession number C_AA057591.1-C_AA057607.1.

## 3. Results

### 3.1. Sequencing Summary

The sequencing process involved collecting 253 avian samples, from which 250 libraries were constructed, resulting in a total of 2,750,483,659 clean data reads. The read counts varied significantly between 1,300,448 and 58,043,413. After filtering out host and rRNA information, a final set of 1,885,277,192 sequencing reads was obtained, constituting an average of 29.05% of the total clean reads.

The analysis using the Standard PlusPF database revealed that 320,347 reads (0.012% of the total clean reads) were attributed to *Virus* by Kraken2 and Bracken. Additionally, 1,338,357,212 reads (48.66% of the total clean reads) were assigned to *Bacteria*. The remaining reads were allocated to *Eukaryota* and *Archaea*.

**Figure 1:**
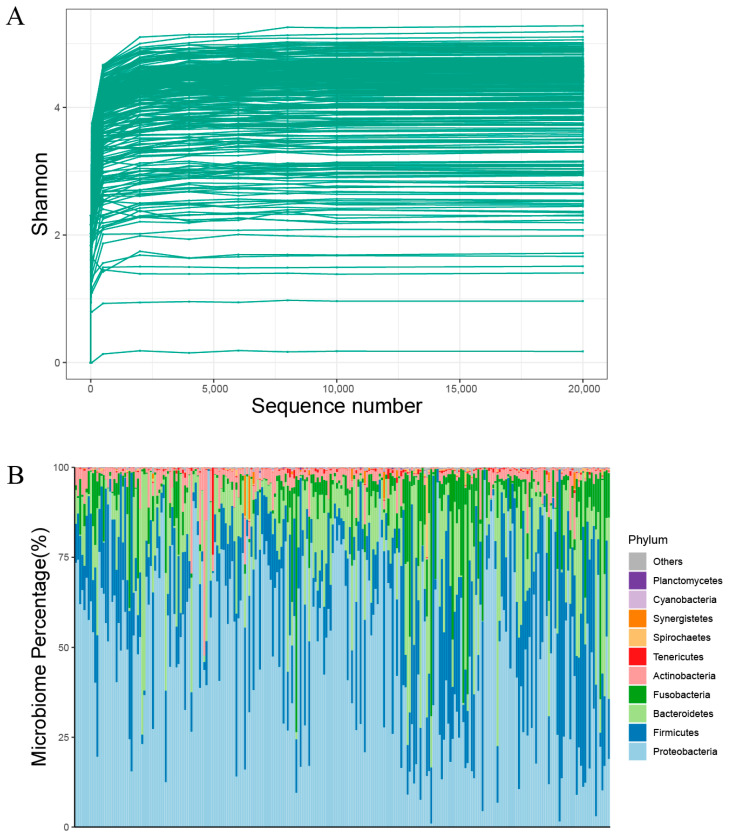
Overview of bacterial abundance after filtering host sequences for 250 samples. (**A**) Rarefaction curve analysis of Shannon index for each sample. (**B**) Composition of bacterial microbiome identified at phylum level.

Rarefaction curves demonstrate the successful recovery of bacterial microorganisms from both fecal and throat swab samples of each bird species (Figure 1). When analyzing the virome separately, a sampling threshold of 100 was set for rarefaction curves, resulting in the retention of curves from only 147 samples. Because many viral rarefaction curves did not reach a peak, we did not remove outliers based on viral rarefaction curves.

### 3.2. Bacterial Microbiome Abundance and Diversity

The taxonomic analysis of bacterial microbiomes in 250 samples utilized Kraken2 and Bracken methodologies. This analysis identified 5600 species across 1526 bacterial genera, spanning 37 phyla, 75 classes, 175 orders, and 403 families (Figure S3).

#### 3.2.1. Bacterial Abundance Analysis

**Figure 2:**
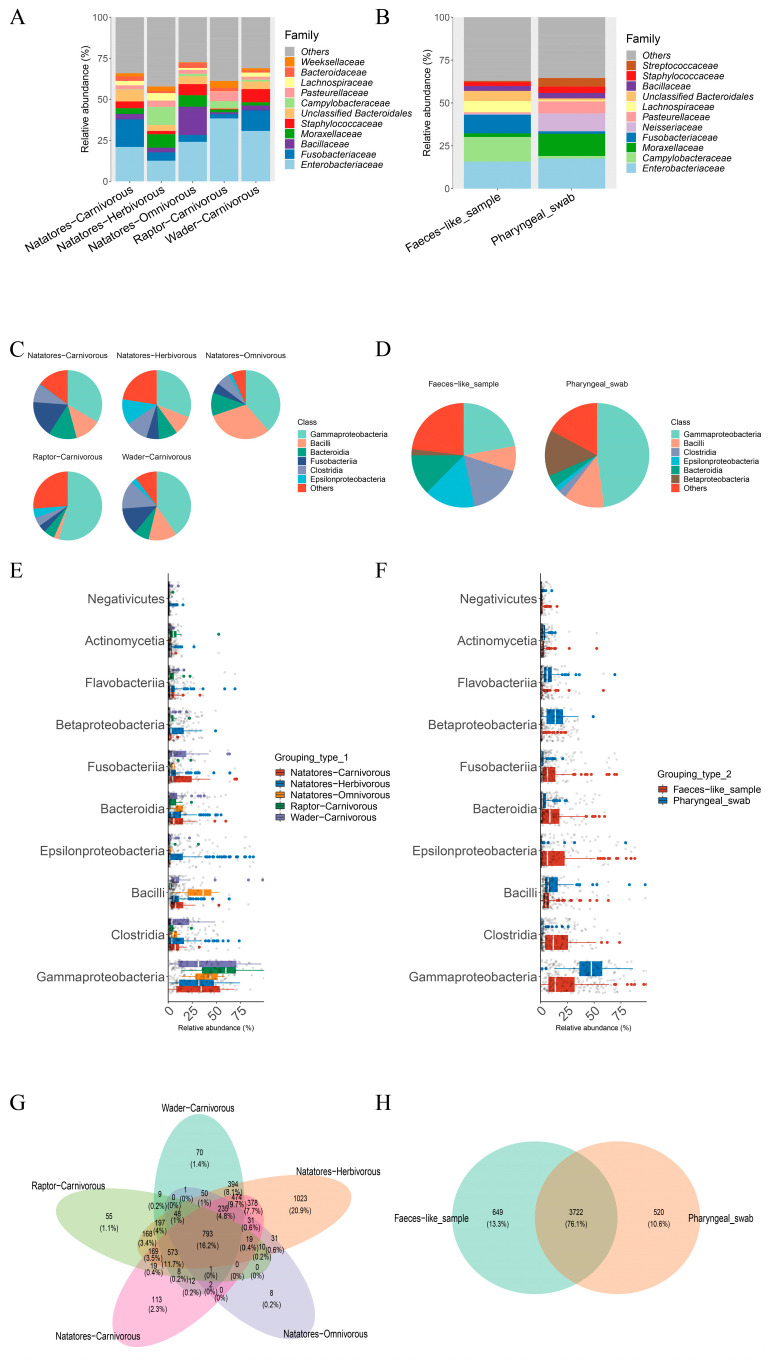
Identification of bacterial microbiome by Kraken2/Bracken in Grouping type 1 (Natatores-Carnivorous, Natatores-Herbivorous, Natatores-Omnivorous, Raptor-Carnivorous, Wader-Carnivorous) and Grouping type 2 (Faeces-like_sample, Pharyngeal_swab). The composition and diversity of the bacterial microbiome identified in Grouping type 1 of (**A**) the bar plot of the top 10 taxa at family level, (**C**) pie plot of the top 6 taxa at class level, (**E**) relative abundance bar plot of the top 10 taxa at class level, and (**G**) Venn diagram. The composition and diversity of bacterial microbiome identified in Grouping type 2 of (**B**) the bar plot of the top 10 taxa at family level, (**D**) pie plot of the top 6 taxa at class level, (**F**) relative abundance bar plot of the top 10 taxa at class level, and (**H**) Venn diagram.

Notably, *Epsilonproteobacteria* exhibited higher abundance within the Natatores-Herbivorous group at the class level, while *Bacilli* predominated in the Natatores-Omnivorous group (Figure 2C,E). *Bacillaceae* notably prevailed in the Natatores-Omnivorous group at the family level. Conversely, the Raptor-Carnivorous group exhibited significantly diminished levels of *Staphylococcaceae*, nearly approaching absence within their microbiome composition (Figure 2A). Additionally, the Venn diagram highlighted shared species among groups, with the Natatores-Herbivorous group hosting the most unique species (1081, 22.9% of all species) (Figure 2G).

At the class level, the feces-like sample group demonstrated increased abundances of *Gammaproteobacteria*, *Clostridia*, *Epsilonproteobacteria*, and *Bacteroidia*, while the pharyngeal swab group displayed elevated levels of *Betaproteobacteria* (Figure 2D,F). Regarding family-level analysis, the feces-like sample group exhibited higher prevalence in *Campylobacteraceae*, *Fusobacteriaceae*, and *Lachnospiraceae*, contrasting with the heightened levels observed in *Moraxellaceae*, *Neisseriaceae*, and *Pasteurellaceae* within the pharyngeal swab group (Figure 2B). Moreover, the Venn diagram revealed shared and unique species among groups, emphasizing distinctions between the feces-like sample group and the pharyngeal swab group (Figure 2H).

#### 3.2.2. Bacterial Diversity Analysis

**Figure 3:**
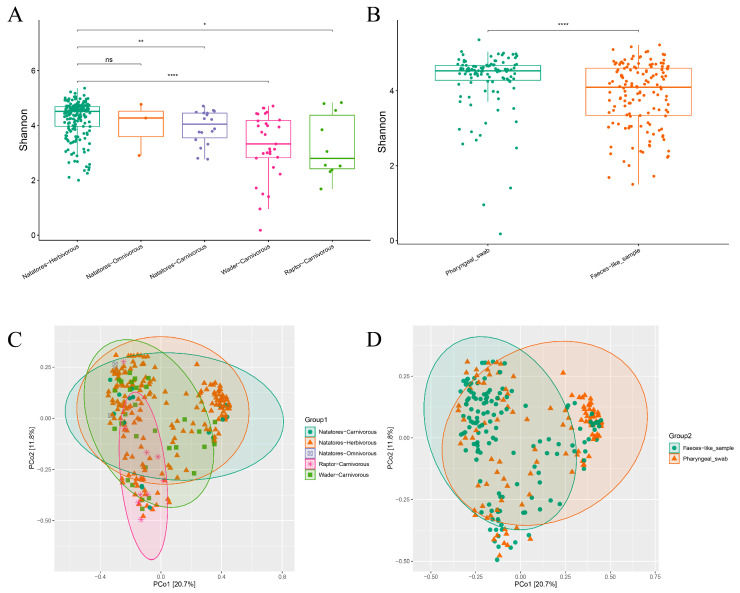
The diversity analysis of bacterial microbiome of Grouping type 1 (Natatores-Carnivorous, Natatores-Herbivorous, Natatores-Omnivorous, Raptor-Carnivorous, Wader-Carnivorous) and Grouping type 2 (Faeces- like_sample, Pharyngeal_swab). (**A**) Boxplot of Shannon index of bacterial microbiome of Grouping type 1; (**B**) boxplot of Shannon index of bacterial microbiome of Grouping type 2; (**C**) PCoA analysis of bacterial microbiome of Grouping type 1 based on Bray–Curtis distance measures with 95% confidence ellipse; (**D**) PCoA analysis of bacterial microbiome of Grouping type 2 based on Bray–Curtis distance measures with 95% confidence ellipse. In the figure, “ns” means *p* > 0.05, “*” means *p* ≤ 0.05, “**” means *p* ≤ 0.001 and “****” means *p* ≤ 0.0001.

Utilizing Kraken2 and Bracken, the bacterial microbiome’s diversity was analyzed, revealing insights into ecological groups and sample types (Figure 3).

The Shannon index indicated significantly higher diversity in the Natatores-Herbivorous group compared to the Raptor-Carnivorous, Natatores-Carnivorous, and Wader-Carnivorous groups (*p* ≤ 0.05). Principal Coordinate Analysis (PCoA) revealed that the disparities within the Natatores-Herbivorous group were primarily manifested along the PCo1 axis, while distinctions among the Natatores-Carnivorous, Raptor-Carnivorous, and Wader-Carnivorous groups were predominantly captured along the PCo2 axis. However, the Natatores-Omnivorous group did not exhibit distinct separation (Figure 3A,C).

In contrast, the feces-like sample group and the pharyngeal swab group were distinguishable according to the PCoA results, indicating significant differences between these two groups (*p* ≤ 0.05) (Figure 3B,D).

### 3.3. Virome Abundance and Diversity

Taxonomic analysis of viruses in 250 samples was performed by Kraken2 and Bracken. A total of 214 species of 59 genera were identified and extended from 10 phyla, 12 classes, 13 orders and 22 families (Figure S4).

#### 3.3.1. Virus Abundance Analysis

**Figure 4:**
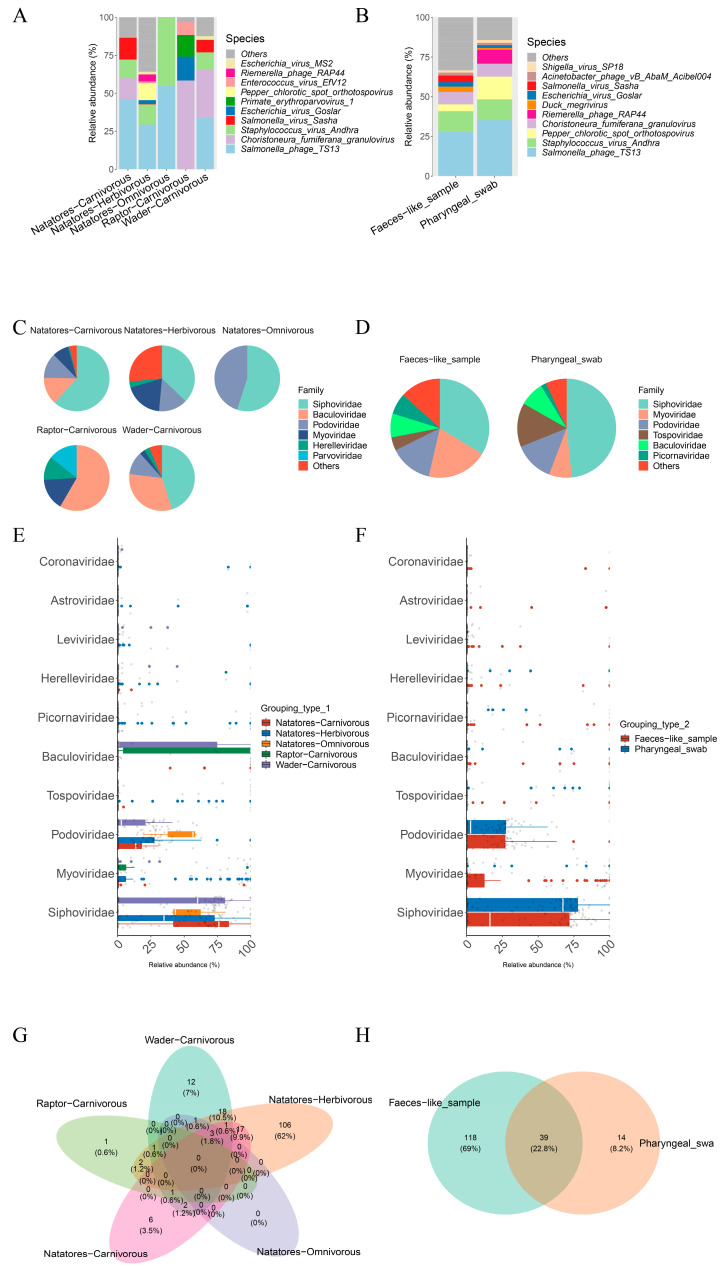
Identification of viruses by Kraken2/Bracken in Grouping type 1 (Natatores-Carnivorous, Natatores-Herbivorous, Natatores-Omnivorous, Raptor-Carnivorous, Wader-Carnivorous) and Grouping type 2 (Faeces-like_sample, Pharyngeal_swab). The composition and diversity of bacterial microbiome identified in different ecological groups of birds of (**A**) the bar plot of the top 10 taxa at species level, (**C**) pie plot of the top 6 taxa at family level, (**E**) relative abundance bar plot of the top 10 taxa at family level, and (**G**) Venn diagram. The composition and diversity of bacterial microbiome identified in Grouping type 2 of (**B**) the bar plot of the top 10 taxa at species level, (**D**) pie plot of the top 6 taxa at family level, (**F**) relative abundance bar plot of the top 10 taxa at family level, and (**H**) Venn diagram.

At the family level, notable abundance was observed for *Parvoviridae*, *Myoviridae*, and *Hereleviridae* in the Raptor-Carnivorous group, while *Siphoviridae* exhibited higher abundance in the other four groups (Figure 4C,E). At the species level, *Salmonella phage TS13* and *Staphylococcus virus Andhra* were the top two species in abundance across the groups, except for the Raptor-Carnivorous group. In contrast, *Escherichia virus Goslar* and *Primate Erythroparvovirus 1* demonstrated the highest abundance in the Raptor-Carnivorous group (Figure 4A). The Venn diagram indicated no common genes among the five groups, with 106 unique species identified in the Natatores-Herbivorous group (Figure 4G).

Family-level assessment revealed higher *Myoviridae* abundance in the feces-like sample group and increased *Tospoviridae* prevalence in the pharyngeal swab group (Figure 4D,F). Specific species such as *Salmonella phage TS13* and *Staphylococcus virus Andhra* exhibited high abundance, while *Pepper chlorotic spot virus* was relatively rare in the feces-like sample group, and *Salmonella virus Sasha* was relatively rare in the pharyngeal swab group (Figure 4B). The Venn diagram highlighted 39 shared species between the two groups, with 118 species exclusive to the feces-like sample group and 14 exclusive to the pharyngeal swab group (Figure 4H).

#### 3.3.2. Viral Diversity Analysis

**Figure 5:**
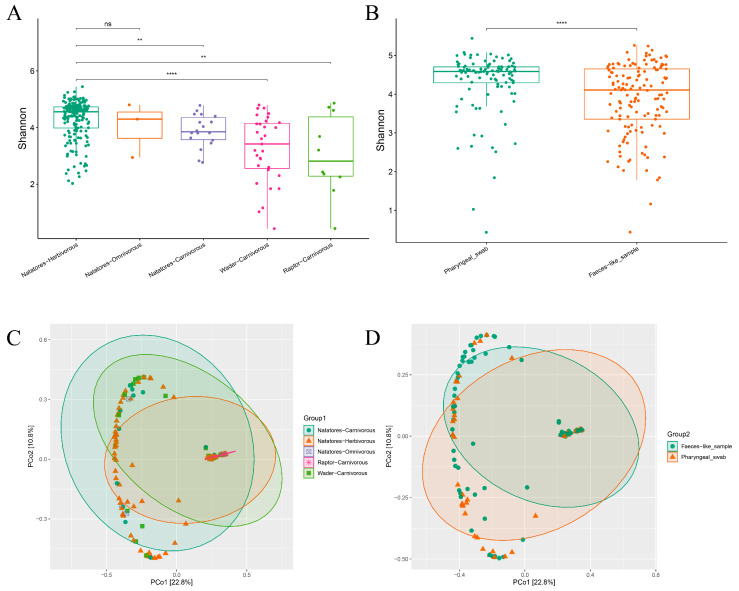
The diversity analysis of viruses of Grouping type 1 (Natatores-Carnivorous, Natatores-Herbivorous, Natatores-Omnivorous, Raptor-Carnivorous, Wader-Carnivorous) and Grouping type 2 (Faeces-like_sample, Pharyngeal_swab). (**A**) Boxplot of Shannon index of viruses of Grouping type 1; (**B**) boxplot of Shannon index of viruses of Grouping type 2; (**C**) PCoA analysis of viruses of Grouping type 1 based on Bray–Curtis distance measures with 95% confidence ellipse; (**D**) PCoA analysis of viruses of Grouping type 2 based on Bray–Curtis distance measures with 95% confidence ellipse. In the figure, “ns” means *p* > 0.05, “**” means *p* ≤ 0.01, and “****” means *p* ≤ 0.0001.

Employing Kraken2 and Bracken, virome diversity analysis indicated intra- and inter-group diversity in Grouping type 1 and Grouping type 2, showcasing distinctions and similarities among ecological groups and sample types (Figure 5).

The Shannon index revealed significantly higher diversity in the Natatores-Herbivorous group compared to the Natatores-Carnivorous, Wader-Carnivorous, and Raptor-Carnivorous groups (*p* ≤ 0.05). PCoA analysis displayed clear separation among all the groups (Figure 5A,C).

Moreover, the median Shannon index indicated significantly lower diversity in the feces-like sample group compared to the pharyngeal swab group (*p* ≤ 0.05) (Figure 5B,D).

It is notable that in our PCoA analysis based on virus reads, a distinct ellipse formed by PCoA characteristic points has emerged. This phenomenon is attributed to the horseshoe effect, a consequence of the relatively small number of virus sequences, thus presenting a limitation in our study. The subsequent PERMANOVA test, utilizing Bray–Curtis distance measures, revealed significant differences (*p* < 0.01) among groups within the clusters categorized by Grouping type 1, and significant differences (*p* < 0.02) among groups within the clusters categorized by Grouping type 2, and the PERMANOVA test result of “Grouping type 1 × Grouping type 2” indicates the interaction between these two variables is not significant (*p* = 0.80) (Table S3).

### 3.4. Taxonomic Biomarker

**Figure 6:**
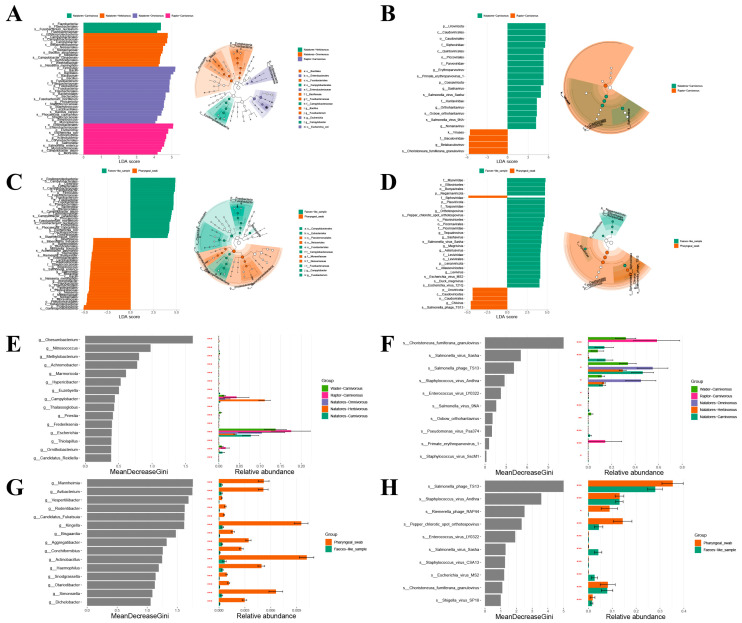
Bacterial and viral signatures significantly different between Grouping type 1 (Natatores-Carnivorous, Natatores-Herbivorous, Natatores-Omnivorous, Raptor-Carnivorous, Wader-Carnivorous) and Grouping type 2 (Faeces-like_sample, Pharyngeal_swab). (**A**) LEfSe analysis identified the most differentially abundant bacterial taxa in Grouping type 1 (LDA > 4). (**B**) LEfSe analysis identified the most differentially abundant viral taxa in Grouping type 1 (LDA > 4). (**C**) LEfSe analysis identified the most differentially abundant bacterial taxa in Grouping type 2 (LDA > 4). (**D**) LEfSe analysis identified the most differentially abundant viral taxa in Grouping type 2 (LDA > 4). (**E**) Random Forest analysis identified the top 15 MeanDecreaseGini value bacterial taxa at genus level in Grouping type 1. (**F**) Random Forest analysis identified the top 10 MeanDecreaseGini value viral taxa at species level in Grouping type 1. (**G**) Random Forest analysis identified the top 15 MeanDecreaseGini value bacterial taxa at genus level in Grouping type 2. (**H**) Random Forest analysis identified the top 10 MeanDecreaseGini value viral taxa at species level in Grouping type 2. In the figure, “*” means *p* ≤ 0.05, “**” means *p* ≤ 0.01 and “***” means *p* ≤ 0.001.

Taxonomic biomarkers and the classification tree between different feeding habits, ecological classifications, and sample types were analyzed using Linear Discriminant Analysis Effect Size (LEfSe) and Random Forest (RF), respectively (Figure 6). The histogram of Linear Discriminant Analysis (LDA) identifies which taxa among all those detected as statistically and biologically differential explain the greatest differences between groups. The MeanDecreaseGini index represented the importance of each genus in distinguishing different groups.

LEfSe analysis revealed the bacterial families with the highest Linear Discriminant Analysis (LDA) values in their respective groups: *Flavobacteriaceae* (Natatores-Carnivorous), *Campylobacteraceae* (Natatores-Herbivorous), *Bacillaceae* (Natatores-Omnivorous), and *Enterobacteriaceae* (Raptor-Carnivorous). At the viral genus level, LEfSe analysis identified *Erythroparvovirus* and *Betabaculovirus* as having the highest LDA values in the Natatores-Carnivorous and Raptor-Carnivorous groups, respectively. RF analysis ranked bacterial genera, highlighting *Escherichia* with the highest MeanDecreaseGini value in the Raptor-Carnivorous group. At the viral species level, the top three species with the highest MeanDecreaseGini values included *Choristoneura fumiferana granulovirus*, *Salmonella virus Sasha*, and *Salmonella phage TS13*.

LEfSe analysis for the feces-like sample group and pharyngeal swab group identified *Campylobacteraceae* and *Moraxellaceae* as the bacterial families with the highest LDA values. At the viral genus level, *Orthotospovirus* and *Chivirus* had the highest LDA values in the feces-like sample and pharyngeal swab groups, respectively. RF analysis indicated the top highest MeanDecreaseGini values for viral species, including *Salmonella phage TS13*, *Staphylococcus virus Andhra*, *Riemerella phage RAP44*, *Pepper chlorotic spot Orthotospovirus*, *Salmonella virus Sasha*, and *Choristoneura fumiferana granulovirus*.

### 3.5. Correlation Analysis of Bacteria and Virus Distribution

**Figure 7:**
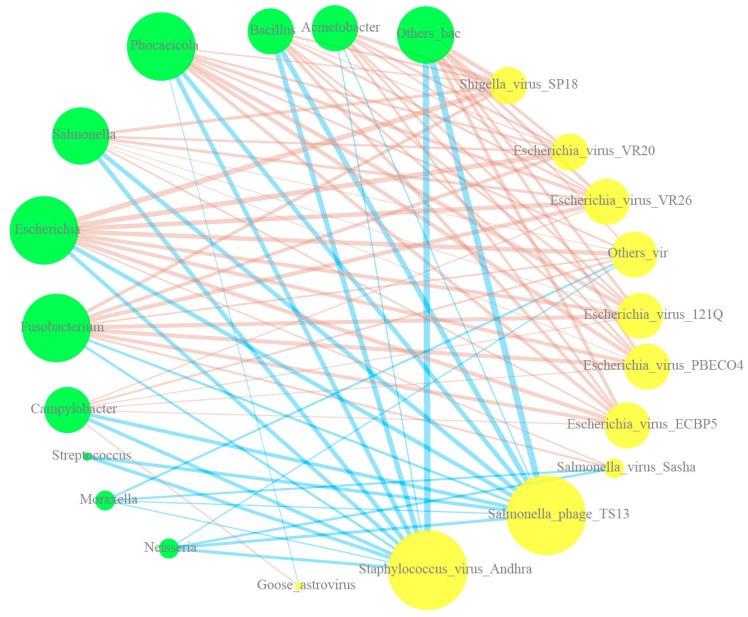
Correlation analysis between the top 10 bacterial genera and the top 10 viral species. All correlation coefficients showed *p*-values below 0.05. Bacterial genera were marked using green circles, and viral species were represented using yellow circles. The size of these circles was proportional to the number of correlated objects. Positive correlations are indicated by brown lines, while negative correlations are indicated by blue lines. The thickness of the lines reflects the correlation value.

The correlations between bacterial microbiome genus-level abundance and viral species-level abundance were analyzed and visualized. Correlations with a *p*-value greater than 0.05 were filtered out, and only the top 10 correlations are shown (Figure 7).

A total of 82 significant correlations were detected, with an average of 7.45 connections. In the virus group, *Salmonella phage TS13* and *Staphylococcus virus Andhra* exhibited 11 negative connections. *Escherichia*, *Fusobacterium*, and *Phocaeicola* displayed 10 connections, with the three bacterial genera showing a negative correlation with *Salmonella phage TS13* and a positive correlation with *Staphylococcus virus Andhra* and *Phocaeicola*-*goose astrovirus*.

### 3.6. Full Genome Sequence Analysis of Viruses

Using Megahit assembled clean data, we obtained 200,819 contigs with a length of longer than 500 bp, summing up to a total sequence length of 205,939,697 bp. Subsequently, 314,892 gene fragments, totaling 177,516,333bp, were identified by prodigal.

Genes were de-redundant based on 95% similarity, resulting in 176,348 non-redundant gene sequences identified as viruses. Eight viral Operational Taxonomic Units (vOTUs) were obtained according to CheckV’s moderate or high-quality results (Table 1).

These sequences were classified under *Astroviridae*, *Caliciviridae*, *Dicistroviridae*, *Picornaviridae*, and *Tombusviridae*. Clean data were recruited for each vOTU using BWA software, and the results with coverage greater than 3× and 50% were listed (Table 2).

#### 3.6.1. Genome Characteristics and Phylogenetic Analysis of Family Astroviridae

**Figure 8:**
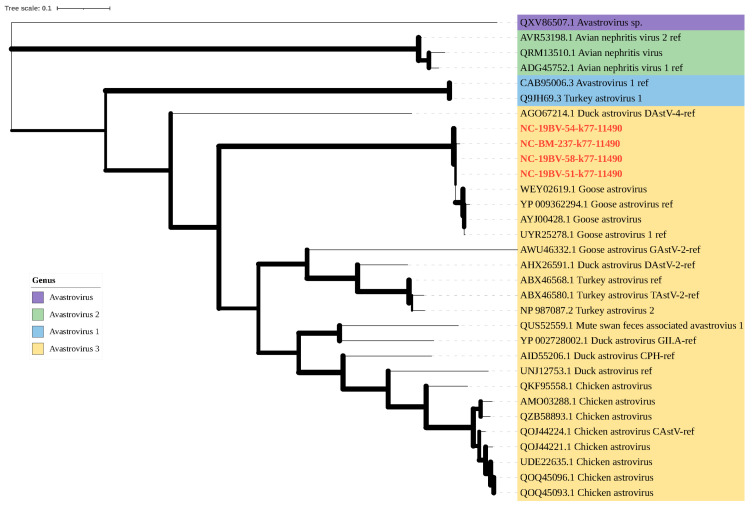
A maximum likelihood phylogenetic tree was constructed using RdRp for the genus Avastrovirus of the Astroviridae family with 1000 bootstrap replicates. The branch widths are calculated from the bootstrap values. The sequences of the viruses that were identified in this study are indicated by the red font. Classification information for genera and species is indicated by the colored background on the branch label.

For the family *Astroviridae*, 29 RdRp sequences of the genus *Avastrovirus* were collected from GenBank. A phylogenetic analysis, incorporating four viral sequences referenced to vOTU k77-11490, showed that the RdRp of k77-11490-associated virus clustered with *goose astrovirus* (*unclassified Avastrovirus*) and *duck astrovirus* (*Avastrovirus 3*). This suggests that *goose astrovirus* and the associated vOTU should be merged into *Avastrovirus 3* of *Astroviridae* (Figure 8).

#### 3.6.2. Genome Characteristics and Phylogenetic Analysis of Order Picornavirales

**Figure 9:**
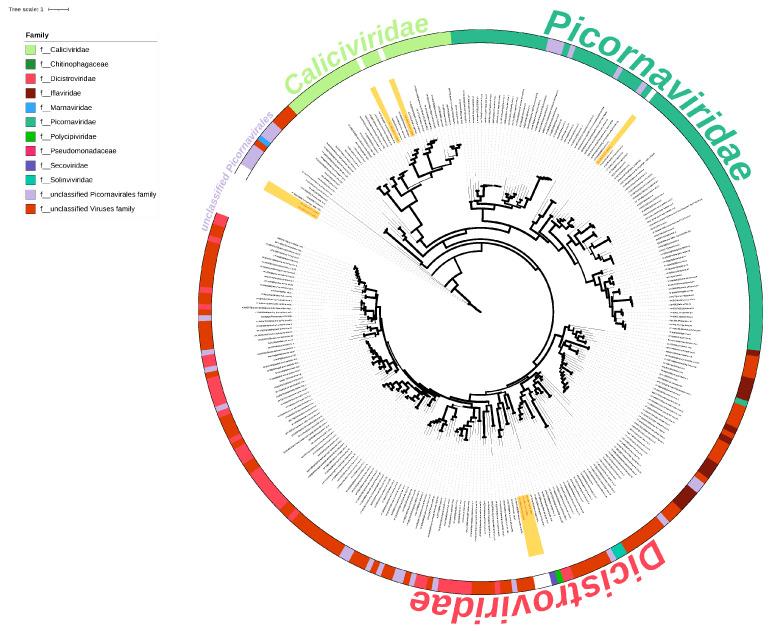
A maximum likelihood phylogenetic tree was constructed using RdRp for Picornavirales with 1000 bootstrap replicates. The branch widths were calculated from the bootstrap values. The sequences of the viruses identified in this study are highlighted in red font with a yellow background. The external annotations indicate annotations of the family level.

Six vOTUs and eight viral sequences of the order *Picornavirales* (*Caliciviridae*, *Dicistroviridae*, *Picornaviridae*) were identified. A phylogenetic analysis, incorporating 289 RdRp sequences of the order *Picornavirales*, revealed topological separations at the family level. The evolutionary tree displayed clustering of RdRps from different vOTUs with *unclassified Picornavirales*, *Caliciviridae*, *Picornaviridae*, and *Dicistroviridae* branches (Figure 9).

#### 3.6.3. Genome Characteristics and Phylogenetic Analysis of Family Tombusviridae

**Figure 10:**
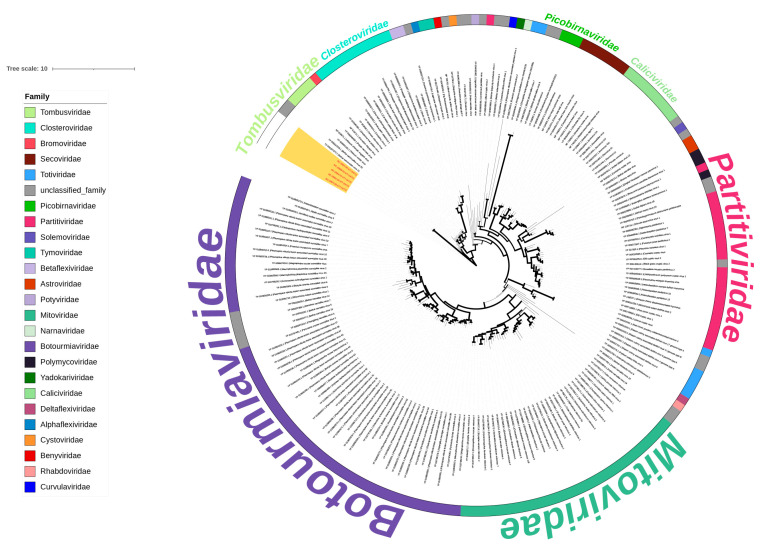
A maximum likelihood phylogenetic tree was constructed using RDRPs for RNA viruses with 1000 bootstrap replicates. The branch widths are calculated from the bootstrap values. The sequences of the viruses identified in this study are highlighted in red font with a yellow background. The external annotations indicate annotations of the family level.

A vOTU (named k77_24772) identified as *Tombusviridae* matched five viral sequences. Phylogenetic analysis of RdRp of the *Riboviria* clade, incorporating 196 viral sequences, indicated that RdRp of vOTU k77_24772 clustered together with the family *Tombusviridae* (Figure 10).

## 4. Discussion

Poyang Lake, the largest freshwater lake in China, plays a crucial role as a transit station for migratory birds, hosting a significant percentage of Siberian cranes, swans, and white-naped cranes during winter. This unique ecosystem facilitates interactions among migratory birds, poultry, and the environment, potentially influencing the transmission of viruses and bacteria. Our pioneering metagenomic study, encompassing endangered bird species, provides comprehensive insights into the bacterial and viral microbiomes within migratory birds at Poyang Lake.

We identified taxonomic biomarkers associated with diverse parameters, including habitat (migrating or wild), feeding habits, ecological classification, and sample types. Notably, *Choristoneura fumiferana granulovirus* emerged as a robust biomarker for carnivorous birds, particularly prevalent in the Wader-Carnivorous, Raptor-Carnivorous, and Natatores-Carnivorous groups. This virus, affiliated with *Baculoviridae*, signifies the dietary preference of carnivorous birds for *Lepidoptera* or *Hymenoptera* insects [28], establishing it as a reliable biomarker.

*Staphylococcaceae* and *Salmonella*, identified in raptors, may influence migratory behavior. *Staphylococcus virus Andhra* and its host, *Staphylococcaceae*, displayed low abundance in the Raptor-Carnivorous group. *Staphylococcaceae* circulate in raptors and cause multicentric septic osteomyelitis and arthritis [29,30]. We hypothesize that *Staphylococcus* infection in raptors may lead to a decision not to migrate or even death during the migration pathway, and non-raptors are more resistant to *Staphylococcus* infections.

*Salmonella* infections, commonly associated with carnivorous and omnivorous birds through the consumption of infected prey [31,32], presented an unexpected scenario in the Raptor-Carnivorous group. Despite the group’s carnivorous nature, the relative abundance of *Salmonella*, *Salmonella phage TS13* and *Salmonella virus Sasha* was notably low. We speculate that *Salmonella* infection may be a serious lethal factor or a factor affecting migratory choices during the migration of *Falconiforms* and *Strigiforms*. Conversely, omnivorous migratory birds displayed resistance to *Salmonella* infections, and *Escherichia* does not seem to play a significant role as a lethal factor or migratory decision influencer for this group.

*Campylobacter*, recognized as a pivotal pathogenic factor in raptors and a potential cause of human infections through contaminated poultry meat [32,33], exhibited high abundance across all groups. Surprisingly, this ubiquity of *Campylobacter* spp. suggests that, contrary to expectations, it may not be a severe lethal factor for migratory birds or a decisive factor influencing migratory selection. Further investigations are required to unravel the nuanced interactions between *Campylobacter* and migratory bird species, shedding light on their resilience to this pathogen during migration.

The differentiation between oral and cloacal samples is facilitated by the prevalence of *Myoviridae* and *Tospoviridae*. Notably, *Myoviridae* is more abundant in feces-like samples, while *Tospoviridae* exhibits higher prevalence in pharyngeal swab samples. Utilizing the Shannon diversity index, we observed diminished diversity in the feces-like sample group compared to the pharyngeal swab group. The comprehensive analysis of the bacterial microbiome and virome composition and diversity underscored distinct features inherent to each sample type.

LEfSe analysis provided valuable insights, identifying *Riemerella anatipestifer* and *Riemerella phage RAP44* as biomarkers of pharyngeal swab samples. Conversely, *Salmonella virus Sasha*, *Escherichia virus MS2*, and *Escherichia virus 121Q* emerged as biomarkers of feces-like samples. These findings contribute to a nuanced understanding of microbial signatures associated with different anatomical sites, enriching our comprehension of the microbiological dynamics within migratory birds.

The acquisition of near-complete viral whole genome sequences in migratory birds at Poyang Lake revealed the predominance of RNA viruses commonly infecting avian species. Notably, we also identified viruses from the *Tombusviridae* family, typically plant-infecting, and the *Dicistroviridae* family, known for insect infections.

*Astroviridae*, causing intestinal diseases in mammals and birds [34], displayed multiple serotypes in humans [35]. Among them, *Avastroviruses* including *Avastrovirus 1* (*turkey astrovirus 1*), *Avastrovirus 2* (*avian nephritis virus 1* and *avian nephritis virus 2*) and *Avastrovirus 3* (*turkey astrovirus 2* and *duck astrovirus 1*) were identified [36].The phylogenetic tree showed that the RdRp of vOTU k77_11490 was close to *goose astrovirus* in unclassified *Avastrovirus*, and *goose astrovirus* and *duck astrovirus* in *Avastrovirus 3* clustered together. In addition, *goose astrovirus* causes kidney damage and even fatal visceral gout in geese, which affects production in farming [37], and *goose astrovirus* has also been shown to cause gout in ducklings and chicks [36]. The *Astroviridae* we identified may have the potential to infect avian species.

The viral genomes identified encompassed sequences from *Caliciviridae*, *Dicistroviridae* and *Picornaviridae*, all belonging to the *Picornavirales*. *Caliciviridae* and *Picornaviridae* are closely related, and the genome structures of both are relatively similar [38]. The *Picornavirales* viral genomes encode nonstructural proteins that vary widely in sequence, and their nonstructural proteins cannot be used to generate meaningful phylogenetic trees, in contrast to the highly conserved RdRp sequences, which are more suitable for phylogenetic analyses [38,39].

Phylogenetic analysis of the RdRp of the *Picornavirales* showed that the two *Caliciviridae* strains clustered in a single branch with the *Chicken calicivirus* of *bavovirus* and the unclassified *Goose calicivirus*. In previous reports, *Chicken calicivirus* mainly infected birds and was a typical avian infection of *Caliciviridae*. *Chicken calicivirus* was first found to infect farmed chickens on German farms causing diarrhea in Germany [40]. *Goose calicivirus* was isolated and characterized in China in 2014 and is related to the genus *Nacovirus* [41]. Their hosts are white-fronted geese and ruddy ducks in this study, belonging to *Anatidae* of the *Anseriformes*, respectively, so we believe that the sequences of vOTU k77_747816 and k77_711556 are *bavovirus*-related viruses and *nacovirus*-related viruses, respectively.

The *Picornaviridae* viruses identified in this study cluster in the *Rabovirus* branch, which is also in the same evolutionary branch as the mammalian and avian viruses of *Enteroviruses* and *Sapeloviruses* [42]. *Rabovirus* is commonly found in Norway rats and primarily infects rodents [43]. The host of the virus is a bean goose of the Anatidae family. Anatidae primarily consume plant stems and leaves, with occasional consumption of mollusks, but they do not prey on rodents. The discovery of *Rabovirus*-associated viruses in a bean goose metagenome may be due to the fact that geese rarely prey on small rodents.

*Tombusviridae* infects plants and causes crop yield reduction [44,45]. The detection of *Tombusviridae* in the digestive tract of avian species has been reported in the past, and the source of *Tombusviridae* has been hypothesized to be the feeding on infected plants [46]. In this study, the hosts of *Tombusviridae* were phytophagous white-fronted geese and bean geese, and omnivorous pied avocets and great crested grebes, respectively. The hosts usually consume the roots and stems of aquatic plants, and the *Tombusviridae* may enter the Intestinal tract after consuming infected aquatic plants. In the RdRp phylogenetic tree of genera within the family *Tombusviridae*, this virus did not cluster with known genera of *Tombusviridae* but was closer to unclassified *Tombusviridae*. Therefore, the tombus-like virus in this study may be a *Tombusviridae* virus belonging to a new genus yet to be classified.

All members of the *Dicistroviridae* infect *Arthropods* [47]. Honeybees infected with this virus can lead to infection of the entire colony [48], and *Dicistroviridae* can also cause severe pathogenic effects in *Arthropods* [49]. The RdRp sequences of the two viral sequences identified as *Dicistroviridae* in the samples were closest to those of the virus isolated from aquatic organisms in Beihai, China (Genebank No. APG78902) [50], which belongs to the *Riboviria*, and is not specifically categorized in the order and suborder classifications. The hosts of vOTU k77_2992 and k77_764108 were bean geese and white-fronted geese, and the detection of *Dicistroviridae* viruses in the digestive tracts of geese has been reported in the past because geese consume aquatic grasses, fish, shrimp, and insects during the breeding season [46]. We hypothesize that the infected *Arthropods* were consumed by natatores. In the present study, bean geese and white-fronted geese were considered to feed on plant foods, but the reality is clearly more complex than simply categorizing the hosts as herbivorous or carnivorous, and bean geese and white-fronted geese occasionally consume arthropod mollusks.

Our study, in line with recent avian metagenomic sequencing efforts, contributes to understanding avian viral compositions [8,51,52,53]. This suggests that avian viruses are easily transmitted between birds. The results from our study contained a large number of *Caliciviridae*, *Dicistroviridae*, and *Picornaviridae*, similar to other avian metagenomic studies [54]. Notably, the variations in amino acid sequence similarities between identified viruses and known strains, particularly the lower similarity viruses (vOTU k77_593432, k77_2992, and k77_764108), warrant further exploration as potential new viruses within the families *Dicistroviridae* and *Picornaviridae*.

Our study has several limitations that should be considered. Firstly, the low viral content in this study makes it difficult to obtain low-abundance viral sequences and retroviruses in the samples. Secondly, we did not use filtration or post-extraction DNase treatments to enrich viral material because we wanted to capture both prokaryotic and viral microbiomes. As migratory birds undergo physical changes during migration, including the development of substantial fat stores and flight muscle hypertrophy and an increased capacity for fat catabolism, our understanding of the regulatory mechanisms behind these changes remains limited, despite the identification of multiple biomarkers.

## 5. Conclusions

Our comprehensive exploration of the bacterial and viral microbiome in wintering migratory birds at Poyang Lake provides a valid and extensive depiction of the microbial landscape. Given the diverse array of wild birds hosting zoonotic diseases, this study sheds light on the rich bacterial and viral microbiome diversity among migratory birds during winter around Poyang Lake. The findings contribute novel insights into the intricate interplay of bird-related bacterial microbiomes and viromes. Several taxonomic biomarkers were identified, enhancing our ability to delineate diverse bird characteristics. The research also uncovered various pathogens and obtained complete viral genome sequences from migratory birds, underlining the potential threat these hosts pose to public health. To deepen our understanding, we recommend a longitudinal study to analyze the temporal dynamics of bacterial and viral microbiomes in migratory birds during their winter residence at Poyang Lake. Additionally, increased seasonal monitoring of migratory birds, coupled with continuous surveillance, is crucial for promptly identifying and addressing potential zoonotic diseases associated with these avian populations. This proactive approach is paramount for effective public health management and prevention strategies.

## Figures and Tables

**Table 1:** The length and classification information of vOTUs close to the full-length genome.

vOTUs Name	Length	Taxon	Most Similar Protein	Identity
k77_11490	7364	*Goose astrovirus*	UYR25277.1	90.13%
k77_24772	4507	*Tombusviridae* sp.	QUS52766.1	83.87%
k77_747816	9651	*Duck calicivirus*	AXF38657.1	66.36%
k77_593432	7935	*Picornaviridae* sp.	AXF38653.1	33.45%
k77_2992	4368	*Dicistroviridae* sp.	QKE55052.1	43.33%
k77_764108	9625	*Dicistroviridae* sp.	QKE55056.1	35.88%
k77_151977	9937	*Picornavirales* sp.	ULF99686.1	77.80%
k77_711556	8454	*Goose calicivirus*	YP_009028574.1	89.33%

**Table 2:** Virus sequences identified in Poyang Lake birds’ samples.

vOTUs	Sample Name	CoverFold	CoverRange	Taxon
k77_151977	NC-19BV-40	8.210828	0.991748	*Picornavirales* sp.
k77_151977	NC-19BV-41	24.42618	1	*Picornavirales* sp.
k77_593432	NC-19BV-58	105.0547	1	*Picornaviridae* sp.
k77_711556	NC-19BV-37	28.88644	1	*Goose calicivirus*
k77_747816	NC-19BV-45	35.35509	1	*Duck calicivirus*
k77_764108	NC-19BM-104	26.01288	0.999688	*Dicistroviridae* sp.
k77_11490	2019-NC-BM-237	3.11692	0.913634	*Goose astrovirus*
k77_11490	NC-19BV-51	355.3012	1	*Goose astrovirus*
k77_11490	NC-19BV-54	3.807985	0.921238	*Goose astrovirus*
k77_11490	NC-19BV-58	409.9043	0.997284	*Goose astrovirus*
k77_24772	NC-19BV-22	29.86843	1	*Tombusviridae* sp.
k77_24772	NC-19BV-51	1212.509	1	*Tombusviridae* sp.
k77_24772	NC-19BV-54	5.285778	0.969825	*Tombusviridae* sp.
k77_24772	NC-19BV-58	49.57932	1	*Tombusviridae* sp.
k77_24772	NC-19BM-61	3.664522	0.957621	*Tombusviridae* sp.
k77_2992	NC-19BV-51	35.86538	1	*Dicistroviridae* sp.
k77_2992	NC-19BV-54	24.92903	1	*Dicistroviridae* sp.

## Data Availability

The clean data and bio-samples can be found in BioProject PRJCA022325 of the National Genomics Data Center (https://ngdc.cncb.ac.cn/, accessed on 18 January 2024). The complete genome sequences of viruses identified in our study were submitted to GenBase and assigned accession number C_AA057591.1- C_AA057607.1.

## Supplementary Materials

The following supporting information can be downloaded at: https://www.mdpi.com/article/10.3390/v16030396/s1, Figure S1: Identification and diversity analysis of the bacterial microbiome was conducted using Kraken2/Bracken among ecological groups of birds (Natatores, Raptor, and Wader), feeding habits (Carnivorous, Herbivorous, and Omnivorous), sample types (Faeces, Cloacal_swab, Pharyngeal_swab), and orders (Anseriformes, Charadriiformes, Ciconiiformes, Falconiformes, Gruiformes, Podicipediformes, and Strigiformes); Figure S2: Identification and diversity analysis of viruses was conducted using Kraken2/Bracken among ecological groups of birds (Natatores, Raptor, and Wader), feeding habits (Carnivorous, Herbivorous, and Omnivorous), sample types (Faeces, Cloacal_swab, Pharyngeal_swab), and orders (Anseriformes, Charadriiformes, Ciconiiformes, Falconiformes, Gruiformes, Podicipediformes, and Strigiformes); Figure S3: Bacterial microbiome composition and abundance visualization of individuals at different levels by Sankey diagrams; Figure S4: Viral microbiome composition and abundance visualization of individuals at different levels by Sankey diagrams. Table S1: Sampling Information Table; Table S2: Venn_Datatable; Table S3: PerMAVONA Result.
