# Supplementary material for: The Multi-Kingdom Microbiome of Wintering Migratory Birds in Poyang Lake, China
Source: Viruses. 2024 Mar 3;16(3):396. doi: 10.3390/v16030396 (PMC10974949; doi:10.3390/v16030396)
Supplement: Supplementary file 1 [file viruses-16-00396-s001.zip › Figure S4.pdf]

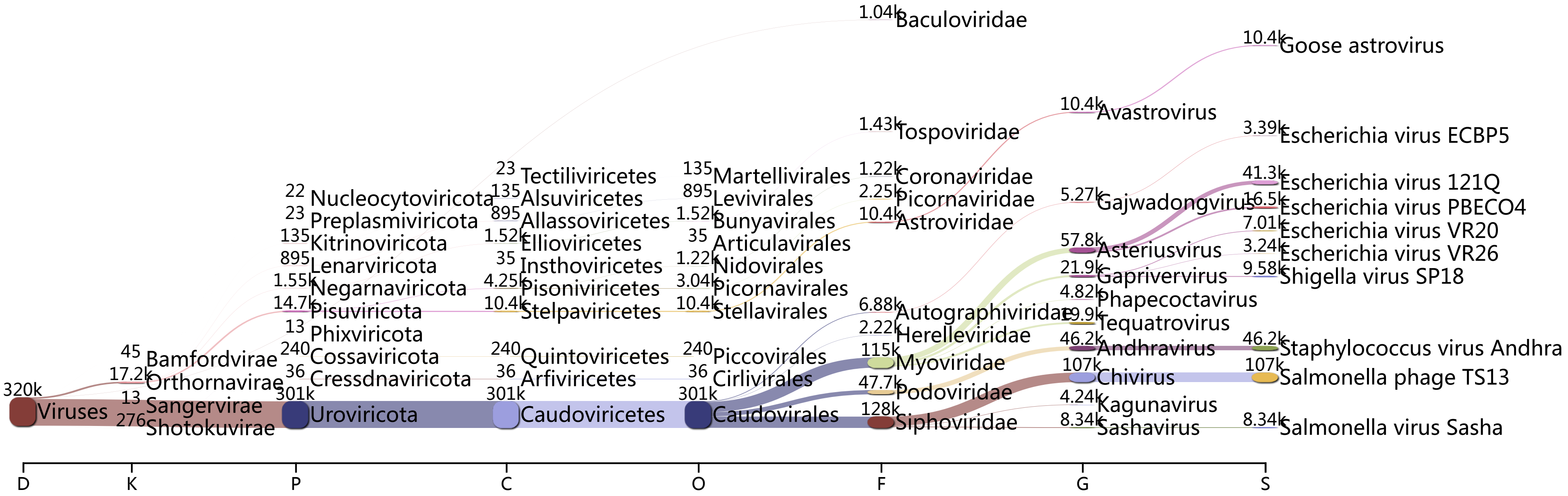

Figure S4. Viral microbiomes composition and abundance visualization of individual at different level by Sankey diagrams. (D – domain, K - Kingdom, P – phylum, C - class, O - order, F - family, G – genus, S - species).
