# Supplementary material for: The Multi-Kingdom Microbiome of Wintering Migratory Birds in Poyang Lake, China
Source: Viruses. 2024 Mar 3;16(3):396. doi: 10.3390/v16030396 (PMC10974949; doi:10.3390/v16030396)
Supplement: Supplementary file 1 [file Figure S2.pdf › Figure S2.pdf]

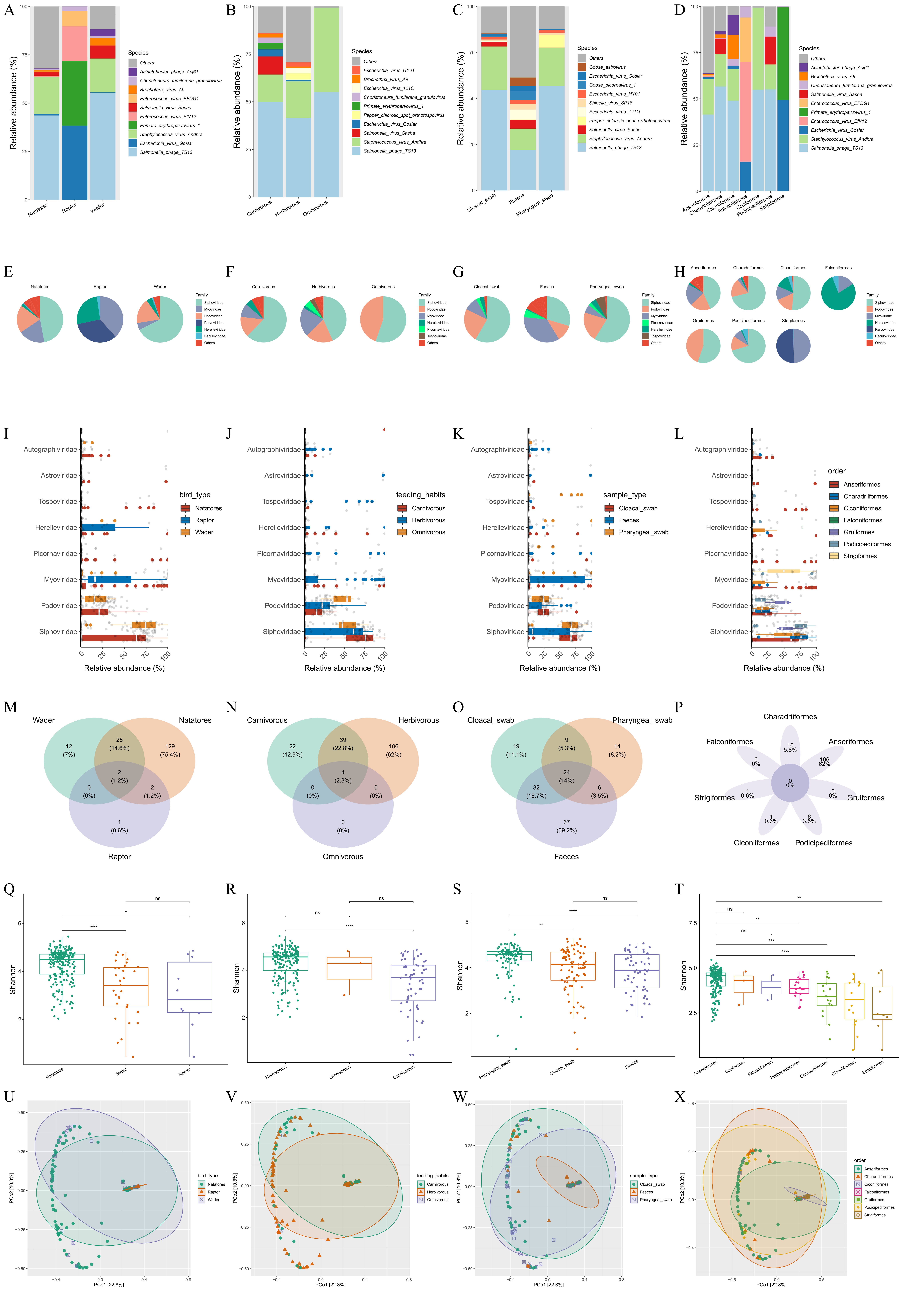

Figure S2. Identification and diversity analysis of viruses was conducted using Kraken2/Bracken among ecological groups of birds (Natatores, Raptor, and Wader), feeding habits (Carnivorous, Herbivorous, and Omnivorous), sample types (Faeces-like_sample, Pharyngeal_swab), and orders (Anseriformes, Charadriiformes, Ciconiiformes, Falconiformes, Gruiformes, Podicipediformes, and Strigiformes). Each column represented a different feature, including bird type (A, E, I, M), feeding habits (B, F, J, N), sample types (C, G, K, O), and orders (D, H, L, P). Each row displayed information on the composition and diversity of viruses identified in different ecological groups of birds using a bar plot of the top 10 taxa at the family level, a pie plot of the top 6 taxa at the class level, a relative abundance bar plot of the top 10 taxa at the class level, a Venn diagram of the genus level, a boxplot of Shannon index, and a PCoA analysis based on Bray-Curtis distance measures with 95% confidence ellipse, respectively. In the figure, "ns" means P > 0.05, "*" means P ≤ 0.05, "***" means P ≤ 0.001, "***" means P ≤ 0.001, "****" means P ≤ 0.0001.
